# Comparison of oral anticoagulation and antiplatelet therapy during the midperiod after percutaneous left atrial appendage closure

**DOI:** 10.3389/fcvm.2025.1637290

**Published:** 2025-11-27

**Authors:** Chang-Yi Li, Lu Zhou, Jing-Rui Zhang, Li-Hong Huang, Li-Zhu Guo, Song-Nan Li, Cai-Hua Sang, De-Yong Long, Jian-Zeng Dong

**Affiliations:** Department of Cardiology, Beijing Anzhen Hospital, Capital Medical University, Beijing, China

**Keywords:** atrial fibrillation, left atrial appendage closure, anticoagulation, antiplatelet, frail

## Abstract

**Background and aim:**

There is a lack of evidence comparing anticoagulation and antiplatelet therapy during the midperiod time after left atrial appendage closure (LAAC) in clinical practice (the “early period” is defined as “45 days” after LAAC for a single procedure of LAAC and “3 months” after LAAC for a combined therapy of LAAC and catheter ablation; we defined the time between the “early period” and 6 months following LAAC as the “midperiod”). Our study aims to assess the safety and effectiveness of different anticoagulant therapies in patients undergoing the LAAC procedure with the WATCHMAN device during the midperiod after LAAC implantation in clinical practice.

**Methods:**

This prospective, single-center cohort study included 374 consecutive patients undergoing percutaneous LAAC with the Watchman device. Patients were divided into two groups: oral anticoagulation (OAC) and antiplatelet therapy (APT). The primary composite endpoint was cardiac mortality, ischemic stroke/ transient ischemic attacks/systemic embolism, and major bleeding events after 6 months following the procedure. The secondary endpoints are cardiovascular death, device-related thrombosis (DRT) events, and each component of the primary endpoint.

**Results:**

The risk of the primary outcome in the APT group and the OAC group had no statistical difference in multivariable Cox regression (adjusted HR = 0.76; 95% CI: 0.40–1.49; *P* = 0.447). The secondary endpoints—including cardiac mortality, cardiovascular death, ischemic stroke/systemic embolism, major bleeding events, and DRT events—did not statistically differ between the two groups.

**Conclusion:**

During the midperiod time after LAAC implantation with the WATCHMAN device, OAC therapy may demonstrate similar safety and efficacy compared with dual antiplatelet therapy.

## Background

Atrial fibrillation (AF) is the most common tachyarrhythmia (1, 2). According to the latest AF guidelines, left atrial appendage closure (LAAC) is recommended as an alternative to oral anticoagulants (OACs) for AF patients with high CHA₂DS₂-VASc scores (3).

However, anticoagulant therapy after LAAC has not been well established and remains controversial. The PROTECT-AF and PREVAIL trials, which compared LAAC to warfarin in patients with contraindications to long-term OAC, are among the most influential randomized controlled trials in this field. In these two trials, OAC was continued for at least 45 days after Watchman device implantation and then replaced by APT only after confirming adequate sealing. Based on these findings, expert consensus recommends OAC for 45 days, followed by dual APT until 6 months, and then single APT lifelong maintenance (4, 5).

Recently, studies focusing on anticoagulant therapy in the early period after LAAC (generally, the “early period” is defined as “45 days” after LAAC for a single procedure of LAAC and “3 months” after LAAC for a combined therapy of LAAC and catheter ablation) have demonstrated that OAC is non-inferior to APT in terms of efficacy and safety(6, 7). Furthermore, some cohort studies have already demonstrated that short-term OAC in the early period following LAAC is safe and tends to be associated with a lower rate of device-related thrombosis (DRT) compared to DAPT (8).

However, there is still a lack of strong evidence comparing antithrombotic therapies during the midperiod time after LAAC (we defined the time between the “early period” and 6 months following LAAC as the “midperiod”). Understanding the comparative effectiveness of OAC vs. APT during the midperiod is crucial for optimizing patient outcomes and establishing evidence-based guidelines, particularly in terms of reducing thromboembolic events and major bleeding risks. To the best of our knowledge, only one study—which recruited 555 patients who were divided into either the standard APT group or the lifelong half-dose non-vitamin K oral anticoagulants (NOAC) group—proved that lifelong half-dose NOAC could significantly reduce the risk of the composite endpoint of DRT, thromboembolism, and major bleeding events compared with the standard APT (9). Despite the well-established early-treatment protocols, the optimal antithrombotic strategy during the midperiod following LAAC remains undefined, creating uncertainty in clinical practice and complicating decision-making for clinicians.

In this study, we hypothesized that a proper prolongation of anticoagulant therapy in the midperiod after LAAC may be non-inferior to APT. Our study aimed to assess the safety and effectiveness of OAC vs. APT in patients undergoing LAAC with the WATCHMAN device during the midperiod time following LAAC implantation in clinical practice.

## Methods

### Study population and clinical follow-up

This study was a prospective, single-centered, hospital-based cohort study that recruited consecutive atrial fibrillation patients undergoing percutaneous LAAC closure with a Watchman device at Beijing ANZHEN Hospital between June 2014 and August 2021.

Inclusion criteria: (1) Age ≥18 years; (2) Non-valvular AF with CHA₂DS₂-VASc score [congestive heart failure, hypertension, age ≥75 years, diabetes mellitus, prior stroke or transient ischemic attacks (TIA) or thromboembolism, vascular disease, age 65–74 years, sex category] ≥2 (men) or ≥3 (women); and (3) Successful WATCHMAN implantation.

Exclusion criteria: (1) Significant peridevice leak (>5 mm) or device-related thrombosis (DRT) at 45-day TEE; and (2) absolute contraindications to APT.

We followed up with most patients who were enrolled into the study via telephone interviews, outpatient interviews, and the WeChat application. Follow-up visits were scheduled 3, 6, and 12 months after treatment and every 6 months thereafter. Data collection was performed by clinical staff who underwent regular training sessions. TEE recordings were evaluated by at least one experienced cardiologist. Interobserver reliability was ensured through periodic calibration of interpretation standards, and all data were subjected to regular auditing for accuracy and completeness. Written informed consent was obtained from each patient, and follow-up information was recorded in the registry database.

### Study group

Generally, the “early period” is defined as “45 days” after LAAC for a single procedure of LAAC and “3 months” after LAAC for a combined therapy of LAAC and catheter ablation. We defined the time between the “early period” and 6 months following LAAC as the “midperiod.”

Eligible patients were assigned to either the OAC or APT group at the discretion of the treating physician, based on individual patient characteristics and risk factors. Generally, all patients received OAC for 45 days after LAAC implantation for a single procedure of LAAC or 3 months after a combined therapy of LAAC and catheter ablation. Then, the patients in the APT group were discharged on dual APT for 6 months, followed by lifelong single APT. Those in the OAC group were discharged on OAC for 6 months and switched to lifelong single APT in the absence of absolute contraindications (the allocation of NOACs or vitamin K antagonists depended mainly on the economic circumstances of the patients).

A *post-hoc* power analysis (*α* = 0.05, *β* = 0.20) indicated that 80% power was required to detect a 7.5% absolute risk reduction in the primary endpoint, assuming an event rate of 15% in the APT group; therefore, if 242 patients were included in the OAC group, 128 patients were needed in APT group in this observational study. The observed effect size (7.7% difference) aligned with the exploratory design of the study. We acknowledge this limitation due to the small sample size and note the requirement for larger, multicenter studies to confirm our findings.

### LAAC procedure

The Watchman device is a self-expanding, nitinol-framed structure ranging in diameter from 21 to 33 mm with fixation barbs and a permeable polyester fabric covering. Implantation was performed through a 12F sheath following a transseptal approach and was guided by fluoroscopy and intracardiac echocardiography (ICE) to verify proper positioning and stability.

### Concomitant catheter ablation procedure

In brief, all ablations used the CARTO3 electroanatomic mapping system. Circumferential pulmonary vein isolation (PVI) was the primary procedure. For paroxysmal AF patients, cavotricuspid isthmus (CTI) ablation was added only if typical atrial flutter (AFL) was diagnosed prior to the procedure; left atrial (LA) roof ablation and mitral isthmus (MI) ablation were rarely performed. For persistent AF, a “2C3L” strategy (PVI plus linear ablation at the LA roof, MI, and CTI) was systematically applied. If AF or organized atrial tachyarrhythmia (OAT) persisted after initial ablation, cardioversion was performed. If unsuccessful or AF immediately recurred, intravenous amiodarone was given before repeat cardioversion. Repeat procedures followed the “2C3L” strategy, targeting conduction gaps and clinical OATs (excluding complex fractionated atrial electrogram ablation). Successful PV isolation and linear block were confirmed by burst pacing from the coronary sinus (200 ms) to induce tachycardia.

### Examination

TEE was performed at 45 days following the single procedure of LAAC or at 3 months following the concomitant catheter ablation and LAAC, as well as at the 6- and 12-month follow-ups to examine whether the LAA closure was adequate and the device stable. A residual peridevice flow of >5 mm observed on 45-day TEE was considered a leak, and a visible large thrombus on the device was considered DRT. TEE was performed using a Philips EPIQ CVx system. Two independent cardiologists assessed the leaks and DRT, with discordance resolved by a third reviewer. TEE recordings were acquired through the WeChat application or at the outpatient face-to-face interview. These recordings were assessed by at least one cardiologist at Beijing ANZHEN hospital.

### Study outcomes

The primary endpoint of this study was to obtain a composite of cardiac mortality, ischemic stroke/TIA/systemic embolism or major bleeding events after 6 months following the procedure. The secondary endpoints were cardiovascular death, DRT events, and each component of the primary endpoint.

### Statistical analysis

Continuous variables were presented as median values with 25th and 75th percentiles, and categorical variables were presented as mean and standard deviations. Baseline characteristics between the OAC group and APT group were assessed via a Fisher’s exact test for dichotomous variables; a two-sample *t*-test was used for normally distributed continuous variables and a two-sample Wilcoxon rank-sum test was used for variables not normally distributed. Clinical outcomes were compared between the two groups using Cox proportional hazards regression models, adjusted for age, gender, type of AF, body mass index, hypertension, coronary artery disease, diabetes mellitus, chronic heart failure, and previous stroke/TIA/systemic embolism. The Kaplan–Meier estimator was used to calculate the time to event rate of the primary and secondary endpoints, whereas log-rank tests were used to compare incidences of the study endpoints between groups. Hazard ratios (HRs) and their 95% confidence intervals (CIs) were calculated to demonstrate the association between the different utilize of OAC and APT with the clinical outcomes. Missing data were minimal and were addressed using multiple imputation techniques to ensure that all available data were used for analysis. Analyses were performed using the STATA software version 17.

## Results

### Baseline characteristic

A total of 374 patients who met the inclusion criteria were enrolled in this cohort with a mean follow-up of 26 ± 18 months; out of these, 132 patients (45.5% concomitant catheter ablation) were in the APT group and 242 (21.1% concomitant catheter ablation) were in the OAC group. There were no patients with absolute contraindication to OAC in both groups. The baseline clinical characteristics were similar between the two groups (Table 1). The patients in the APT group had a mean age of 67 years, 22% were women, and 68.2% were diagnosed as having persistent AF; the patients in the OAC group had a mean age of 67 years, 25.6% were women, and 67.0% were diagnosed as having persistent AF. The difference between the two groups was not statistically significant. The difference between the two groups relating to left ventricular ejection fraction (LVEF), CHA2DS2-VASc scores, HAS-BLED score, and comorbidities such as hypertension, diabetes mellitus, chronic heart failure, and coronary artery disease was not statistically significant.

**Table 1:** Baseline characteristics of study population.

Parameter	OACs group (*n* = 242)	APT group (*n* = 132)	*P*-value
Age, years	67 (60.74)	67 (63,73)	0.291
Female, *n* (%)	62 (25.6)	29 (22.0)	0.452
Body mass index, kg/m^2^	27.5 (24.0,28.1)	27.5 (24.0,28.1)	0.361
Type of AF, *n* (%)			0.699
Persistent	162 (67.0)	90 (68.2)	
CHA2DS2-VASc score (continuous)	4 (3.5)	4 (3.5)	0.932
CHA2DS2-VASc score (categorical), *n* (%)			0.754
2	41 (16.9)	26 (19.7)	
3	50 (20.7)	31 (23.5)	
4	56 (23.1)	29 (22.0)	
≥5	95 (39.3)	46 (34.9)	
HAS-BLED score (continuous)	1.9 (1.3)	1.9 (1.2)	0.497
HAS-BLED score (categorical), *n* (%)			0.333
0	12 (5.0)	7 (5.3)	
1	81 (31.5)	46 (34.9)	
2	87 (36.0)	48 (36.4)	
3	54 (22.3)	24 (18.2)	
4	8 (3.3)	4 (3.0)	
≥5	0	3 (2.3)	
EF, %	57 (58.65)	57 (56.65)	0.200
Stroke/TIA, *n* (%)	84 (73.0)	56 (65.1)	0.278
Diabetes, *n* (%)	74 (30.6)	37 (28.0)	0.637
Hypertension, *n* (%)	176 (72.7)	97 (73.5)	0.904
CAD, *n* (%)	58 (24.0)	42 (31.8)	0.113
Heart failure, *n* (%)	24(9.9)	19(14.4)	0.235

Values are given as mean ± SD or *n* (%). AF, atrial fibrillation; TIA, transient ischemic attack.

During the midperiod following LAAC, in the OAC group (*n* = 242), all patients exclusively received direct oral anticoagulants (DOACs); no patients received warfarin. The specific DOACs used were rivaroxaban (*n* = 159, 65.7%), edoxaban (*n* = 53, 21.9%), and dabigatran (*n* = 30, 12.4%). In the APT group (*n* = 132), all patients received dual antiplatelet therapy (DAPT). In particular, 81 patients (61.4%) received aspirin in combination with clopidogrel, and the remaining 51 patients (38.6%) received aspirin in combination with ticagrelor. No other antithrombotic regimens were used in the APT group. We appreciate the opportunity to clarify these details.

### Primary outcome

**Figure 1:**
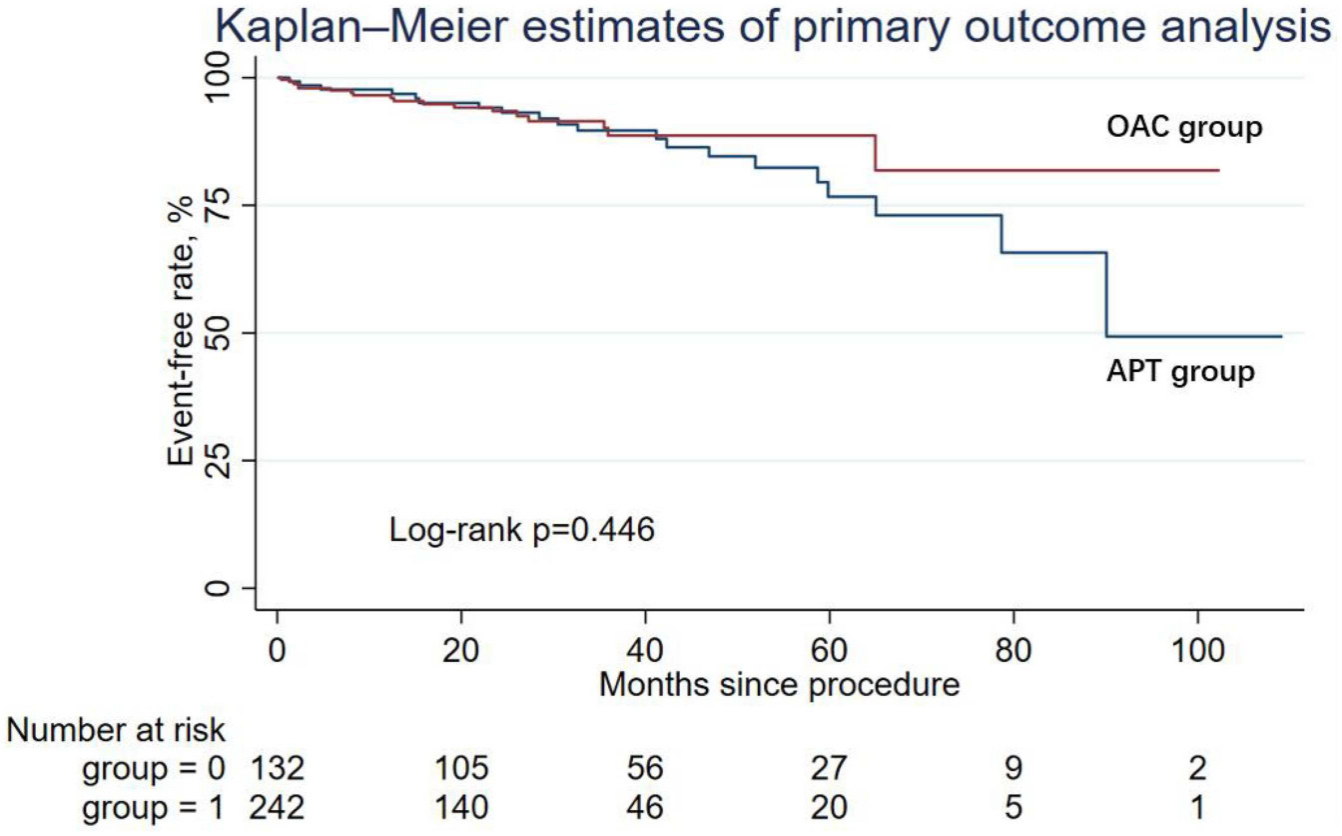
Kaplan-Meier curve showing primary outcome analysis for two groups: OAC (red line) and APT (blue line). The log-rank *p*-value is 0.446.

There were 20 primary endpoints in the APT group and 18 cases in the OAC group, with rates of 15.15% and 7.44%, respectively, but the difference was not statistically significant. The Kaplan–Meier estimator of the primary outcome is shown in Figure 1. The log-rank test of the composite primary outcome was not significantly different between the APT group and the OAC group. Table 2 shows the primary endpoint in the univariable analysis and multivariable analysis. According to multivariable Cox regression, the risk of the primary outcome did not differ significantly between the APT and OAC groups (adjusted HR = 0.67; 95% CI: 0.33–1.36; *P* = 0.271).

**Table 2:** The incidence rates, the primary endpoints, and the secondary endpoints in the univariable and multivariable Cox regression analysis.

Outcome	Incidence rate (%)		Unadjusted HR (95% CI)	*p*	Adjusted HR (95% CI)	*p*
	APT group	OAC group				
Primary endpoint	15.15	7.44	0.78 (0.40, 1.49)	0.447	0.67 (0.33, 1.36)	0.271
Cardiac mortality	6.82	1.65	0.59 (0.17, 2.04)	0.253	0.64 (0.14, 2.90)	0.565
Ischemic stroke/systemic embolism	3.03	1.24	0.44 (0.10, 2.00)	0.289	0.37 (0.06, 2.35)	0.295
Major bleeding events	1.52	0.83	0.62 (0.09, 4.43)	0.637	0.50 (0.03, 7.10)	0.608
DRT events	2.27	0.83	0.84 (0.14, 5.23)	0.854	0.79 (0.07, 8.99)	0.847

In multivariable Cox analysis, we adjusted for age, gender, type of AF, body mass index, hypertension, coronary artery disease, diabetes mellitus, chronic heart failure, and concomitant AF ablation.

### Secondary outcomes

**Figure 2:**
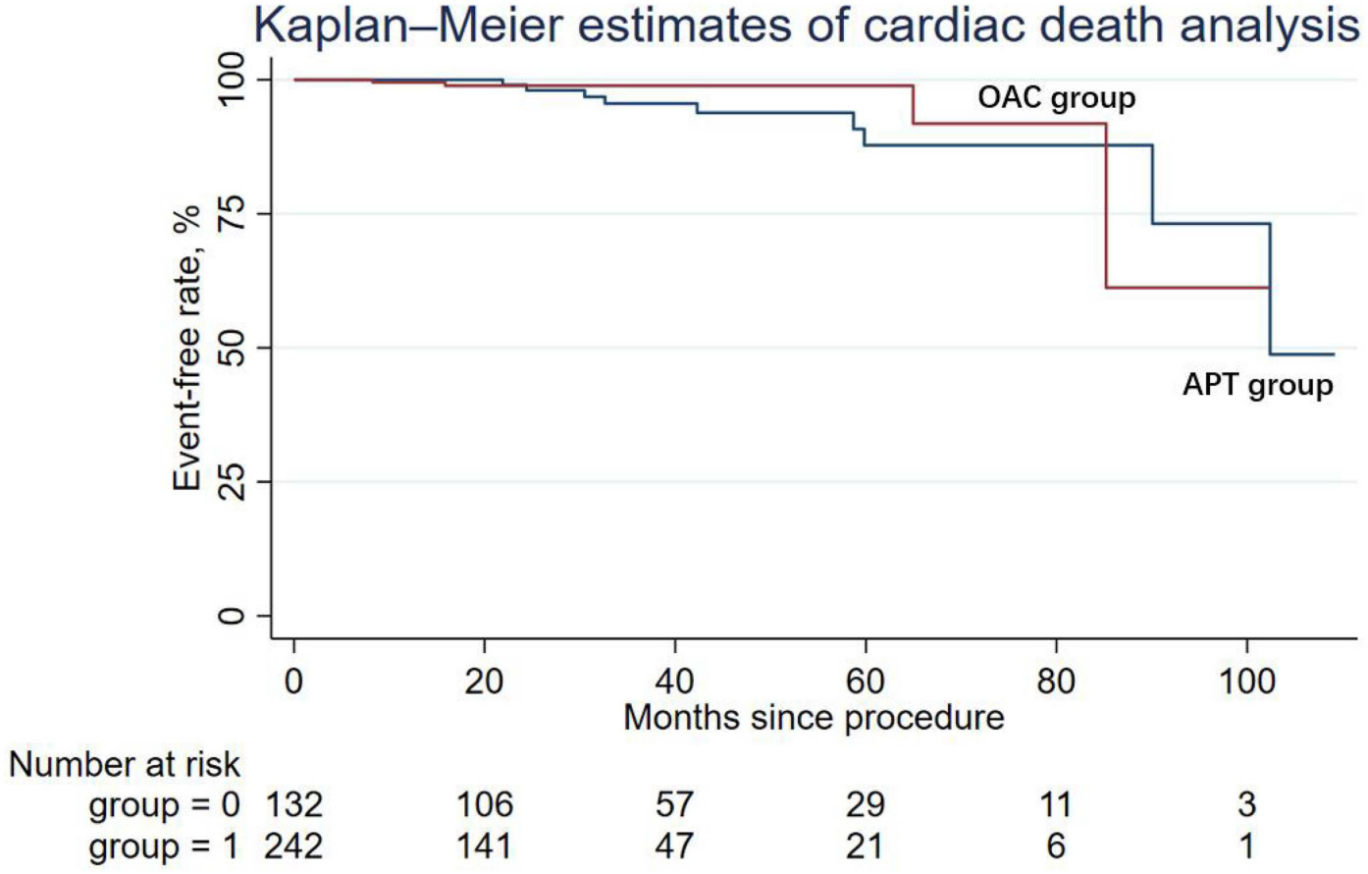
Kaplan-Meier survival curve comparing event-free rates for cardiac death between the OAC and APT groups.

**Figure 3:**
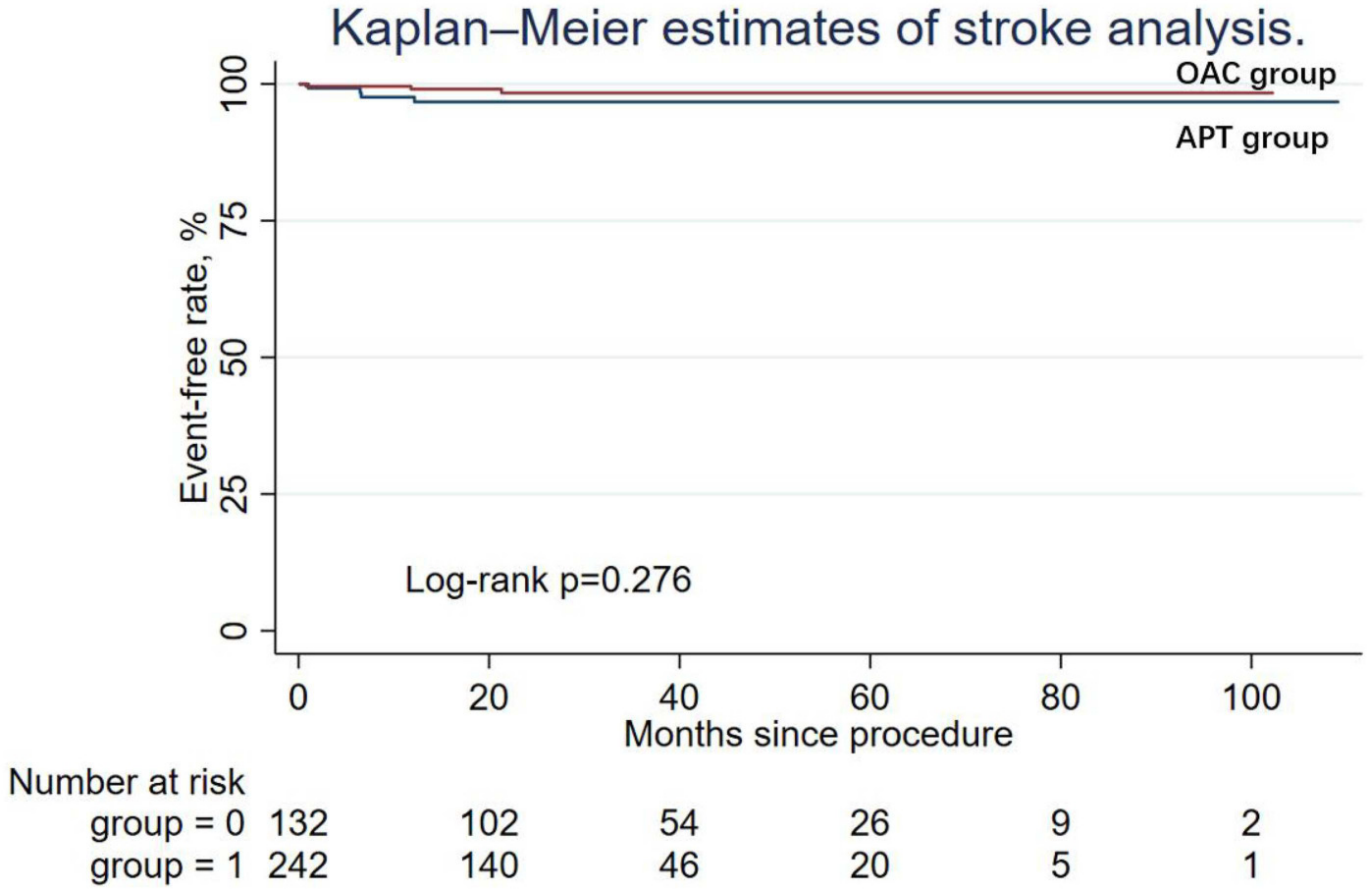
Kaplan-Meier survival curve comparing event-free rates for stroke between the OAC and APT groups.

**Figure 4:**
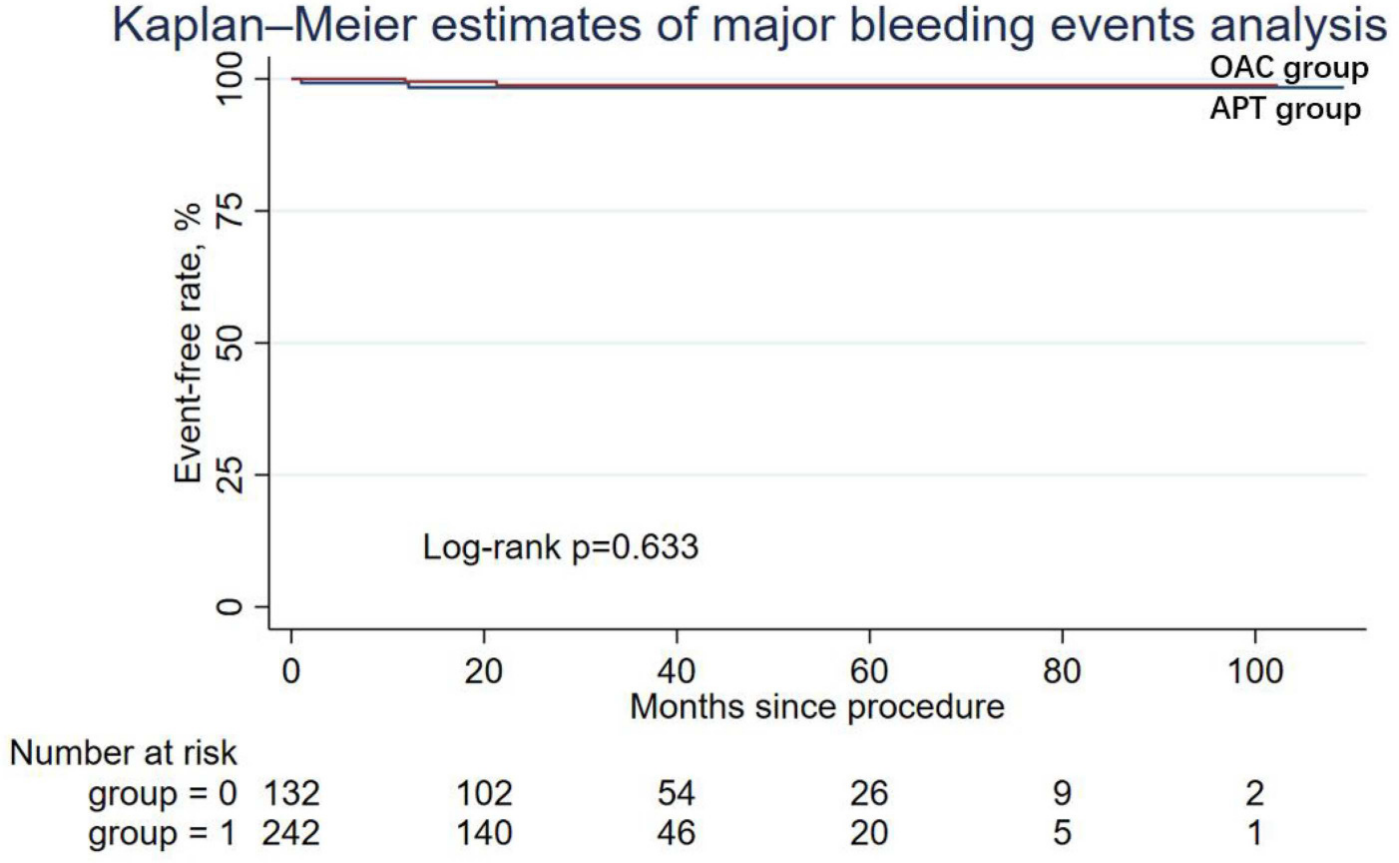
Kaplan-Meier survival curve comparing event-free rates for major bleeding events between the OAC and APT groups.

**Figure 5:**
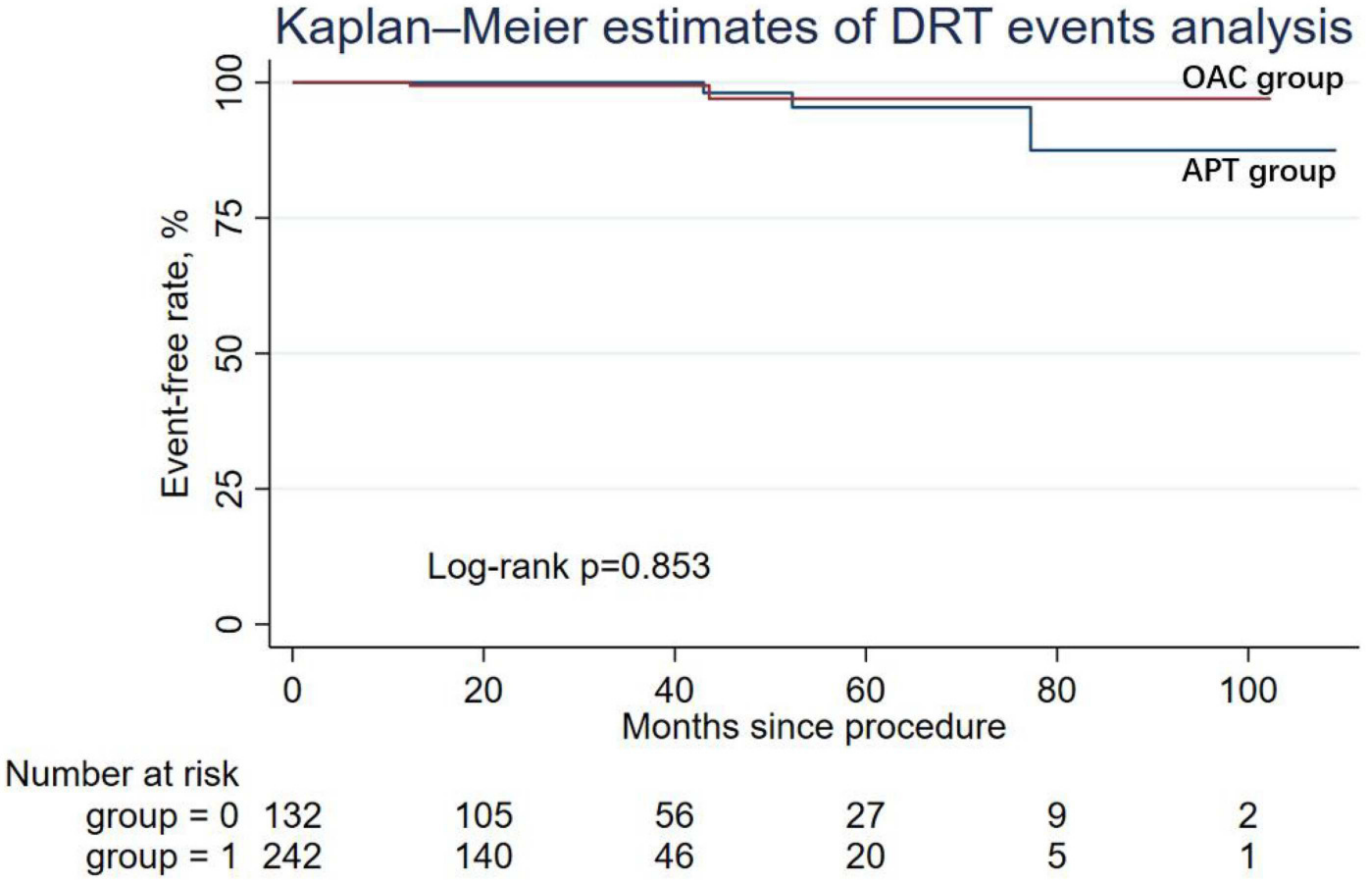
Kaplan-Meier survival curve comparing event-free rates for DRT events between the OAC and APT groups.

During the follow-up period, nine patients (6.82%) in the APT group and four patients (1.65%) in the OAC group experienced cardiac death, but the difference was not statistically significant in Cox regression (adjusted HR = 0.64; 95% CI: 0.14–2.90; *P* = 0.565). Similarly, other secondary endpoints, including ischemic stroke/systemic embolism, major bleeding events (adjusted HR = 0.50; 95% CI: 0.03–7.10; *P* = 0.608), and the DRT events (adjusted HR = 0.79; 95% CI: 0.07–8.99; *P* = 0.847), were not statistically different between the two groups. The Kaplan–Meier estimator and log-rank test for each secondary endpoint showed an identical result (Figures 2–5); the details of each secondary endpoint are shown in Table 2.

## Discussion

This was a prospective, single-center cohort study. The major result of this study is that there is no statistical difference between OAC and APT in cardiac death events during the midperiod following LAAC implantation with the WATCHMAN. OAC therapy during the midperiod after LAAC implantation with the WATCHMAN device may demonstrate better safety and efficacy effects compared with dual antiplatelet therapy.

Expert consensus on LAAC and guidelines on atrial fibrillation—citing the results of the PROTECT-AF and PREVAIL trials—recommend the use anticoagulant drugs for 45 days and then continued use of dual antiplatelet drugs for up to 6 months (3, 10). However, the PROTECT-AF and PREVAIL trials were carried out before NOAC was widely used (NOACs were used in only 5% of the patients in the OAC group) (4, 5). Therefore, there was some research focus on comparing the effects of NOAC and APT following implantation of the LAAC device. A meta-analysis of 10 cohort studies involving 2440 patients proved that NOAC was associated with a lower rate of major bleeding events, and all bleeding events were compared with warfarin following WATCHMAN implantation; there were no significant differences between patients receiving NOAC and those receiving warfarin in terms of thromboembolism, cardiac mortality, DRT, and PDL of >5 mm (11).

Recently, several studies have shown the identical benefits of OAC and APT as immediate therapy after LAAC with the WATCHMAN device. A study conducted by Lars Sondergaard et al.—which included 1,018 patients in the OAC group and 509 patients in the APT group (OAC group: 45-day OAC after implantation followed by 6-month single or dual APT; APT group: received APT for variable durations)—found that in the early period after LAAC, OAC therapies did not demonstrate poorer safety and efficacy effects than APT (12). A sub-study of the EWOLUTION study, which included 998 patients with successful WATCHMAN implantation, showed that the risk of a composite ischemic endpoint of stroke, transitory ischemic attack, systemic embolism and device thrombus, or the major bleeding was not significantly different between the patients undergoing dual APT, single APT, OAC, or no therapy in the early period and long period (6 months) following LAAC (13). Another multicenter cohort study included 592 patients who underwent LAAC and received either dual APT or OAC (477 in the dual-APT group and 115 in the OAC group) for a short period following LAAC; the study demonstrated that the composite outcome of death, stroke, and bleeding was not significantly different between the two groups (14). Upon referring to these studies, the instructions for use in European Conformity marked countries were amended, and both antithrombotic regimens were adopted for use in the short period following LAAC with the WATCHMAN device (14).

However, there is still a lack of evidence comparing the effect of anticoagulation and antiplatelet therapy during the midperiod following LAAC. A study by Domenico G. Della Rocca on 555 patients, who were categorized into two groups (standard APT group: guideline recommended therapy which could be briefly described as taking 45 days NOAC and then dual antiplatelet drugs for up to 6 months, then switching to single antiplatelet drug for life; lifelong half-dose NOAC group: half-dose NOAC plus aspirin 81 mg for 45 days after Watchman implantation and lifelong half-dose NOAC monotherapy thereafter), demonstrated that after successful LAAC implantation with the Watchman device, midperiod and long-period half-dose NOAC could significantly reduce the risk of the composite endpoint of DRT, thromboembolism events, and major bleeding compared with a standard, antiplatelet-based, antithrombotic therapy. Although our findings showed numerically lower DRT rates in the OAC group compared with the APT group, the difference was not statistically significant. This result is generally consistent with the observations reported by Della Rocca et al. We believe the main reason for our non-significant result, which diverged from that of Della Rocca, was due to the difference in patient populations (e.g., baseline risk factors, race, and comorbidities of patients) in terms of treatment efficacy (9).

The results of our study showed that the risk of the primary composite endpoint of cardiac mortality, ischemic stroke/TIA/systemic thromboembolism, and major bleeding events was not significantly different; however, the HR of the Cox regression showed a trend of superiority of the OAC group. This finding was in line with the research results of Domenico G. Della Rocca. As the baseline CHA2DS2-VASc and HAS-BLED scores were similar between the APT group and OAC group, the risk of cardiac mortality, ischemic stroke/TIA/systemic thromboembolism, or major bleeding events for the patients who underwent OAC therapy was similar to the risk for patients who underwent APT. And this result reported in our study was also in accordance with previous studies focused on the early period antithrombosis strategies post LAAC the risk of cardiac mortality, ischemic stroke/TIA/systemic thromboembolism or major bleeding events with the use of OAC compared to APT during the midperiod after LAAC implantation. The results of this study indicated that OAC therapy had a superior trend of safety and efficacy compared to APT, including the endpoint of cardiac mortality, cardiovascular death, ischemic stroke/TIA/systemic thromboembolism, and major bleeding events. Although the hazard ratio for the primary composite outcome showed a trend favoring OAC (adjusted HR = 0.67; 95% CI: 0.33–1.36), the difference was not statistically significant. Clinically, this suggests that OAC may provide similar or slightly superior outcomes compared to APT, but larger studies are needed to confirm this trend. We hypothesized that the results may provide new evidence for OAC utilization after the early period following LAAC implantation. Some studies suggest that patients taking one pill show 10%–15% higher adherence rates than those taking two separate pills per day. As a result, taking OAC rather than APT may have a better patient compliance rate. Compared dual APT, the usage of OAC may have a better compliance rate.

In addition, it has been shown that compared with APT, anticoagulant therapy is more effective in preventing DRT. Earlier research suggested that complete endothelialization occurred within 45 days following LAAC; however, a recent meta-analysis showed that 85% of DRT occurred after 45 days following LAAC (15, 16). Therefore, it is of clinical significance to study the antithrombotic regimen during the midperiod following LAAC. Fauchier et al. and a *post-hoc* analysis of PROTECT AF by Main et al. showed the non-inferior effect of OAC compared to dual APT on DRT risk following LAAC (DRT incidence in the OAC group and the APT group: 8% vs. 79%) (17, 18). These findings were in accordance with the results relating to DRT in our study. Previous research showed that DRT may occur after more than 1 year following LAAC implantation, which may have resulted in the negative DRT event rate comparing the OAC group and APT group of our study and other previous studies (18).

## Limitation

There are some limitations to our study: First, this was a non-randomized cohort study and had the limitations and biases inherent to an observational, retrospective study. There may be some unknown confounders which may influence the accuracy of the study results. Second, our study only recruited patients who used a Watchman device. This makes it difficult to generalize the results of our research to other LAAC devices. Third, the small sample size is another limitation of this study, as it may have led to an overinterpretation of the results. We believe that larger multicenter clinical trials are needed to better solve this problem.

## Conclusion

In conclusion, OAC therapy during the midperiod following LAAC implantation with the WATCHMAN device may offer comparable safety and efficacy to APT. Further multicenter randomized trials are needed to confirm these findings and identify patient-specific factors that influence treatment response.

## Data Availability

The raw data supporting the conclusions of this article will be made available by the authors, without undue reservation.
